# Exploring tear fluid biomarkers and the ocular surface in thyroid eye disease

**DOI:** 10.1111/aos.17556

**Published:** 2025-07-07

**Authors:** Mikael Thomassen Neset, Roy Miodini Nilsen, Kristian Løvås, Kathrine Halsøy, Håkon Reikvam, Ann‐Elin Meling Stokland, Grethe Åstrøm Ueland, Anette S. B. Wolff, Hans Christian D. Aass, Sjur Reppe, Eystein Sverre Husebye, Eyvind Rødahl, Tor Paaske Utheim, Hans Olav Ueland

**Affiliations:** ^1^ Department of Ophthalmology Haukeland University Hospital Bergen Norway; ^2^ Department of Clinical Medicine University of Bergen Bergen Norway; ^3^ Faculty of Health and Social Sciences Western Norway University of Applied Sciences Bergen Norway; ^4^ Department of Medicine Haukeland University Hospital Bergen Norway; ^5^ Department of Clinical Science University of Bergen Bergen Norway; ^6^ Department of Medicine Stavanger University Hospital Stavanger Norway; ^7^ Department of Medical Biochemistry Oslo University Hospital Oslo Norway; ^8^ Department of Plastic and Reconstructive Surgery Oslo University Hospital Oslo Norway; ^9^ Department of Ophthalmology Oslo University Hospital Oslo Norway

**Keywords:** CD40 ligand, chemokine (C–C motif) ligand 2, dry eye disease, Graves' disease, ocular surface disease, thyroid eye disease

## Abstract

**Purpose:**

To examine ocular surface changes and inflammatory tear fluid biomarkers in patients with thyroid eye disease (TED).

**Methods:**

We included 106 Graves' disease (GD) patients (36 without TED, 32 with active and 38 with inactive TED) and 106 age‐ and sex‐matched healthy subjects for ophthalmological evaluation, including ocular surface status and Meibomian gland function. Tear fluid was analysed for 40 inflammatory biomarkers by a Luminex multiplex bead assay. The parameters were compared across subgroups.

**Results:**

GD patients with TED had significantly higher median (min–max) Ocular Surface Disease Index (OSDI) score than GD patients without TED, with a median score of 31.4 (0–86.4) compared to 7.3 (0–45.8) (*p* < 0.01). Eleven of 50 patients with moderate‐to‐severe and sight‐threatening TED had a meibum quality score above seven, compared to none of the 20 patients with mild TED (*p* = 0.027).

Tear fluid levels of chemokine (C–C motif) ligand 2 (CCL2) were significantly (*p* = 0.003) higher in GD patients compared to healthy subjects. CD40 ligand (CD40L) and CCL2 were higher in GD patients with TED compared with GD patients without TED (*p* = 0.002 and 0.013, respectively). As a combined biomarker to distinguish between GD patients with and without TED, OSDI score together with tear fluid levels of CCL2 and CD40L produced an area under the receiver operating characteristic (ROC) curve of 0.80, with sensitivity of 0.69 at a fixed specificity of 0.80.

**Conclusions:**

Our findings demonstrate increased tear fluid levels of CD40L and CCL2 in patients with TED, indicating their potential as diagnostic biomarkers. Increased ocular discomfort in patients with TED could be related to impaired meibum quality.

## 1 INTRODUCTION

Thyroid eye disease (TED) is the most common extrathyroidal manifestation of Graves’ disease (GD), with a yearly incidence of 3.3/100 000 in women and 0.9/100 000 in men (Abraham‐Nordling et al., 2011). It affects about 40% of GD patients (Chin et al., 2020). The disease often results in proptosis, strabismus, eyelid retraction and disfigured appearance. Even mild manifestations influence daily life activities and quality of life (Kahaly et al., 2005; Ponto et al., 2011; Wiersinga, 2012). Ocular surface involvement, including keratopathy and unstable tear film, is a common finding in TED (Nowak et al., 2005), frequently associated with reduced tear production, incomplete blinking and Meibomian gland dysfunction (Kim et al., 2015; Park & Baek, 2019; Satitpitakul et al., 2021).

TED has two phases: an initial, active phase with orbital inflammation, followed by a chronic, inactive phase with fibrosis. The orbital autoimmune reaction is initiated by thyrotropin receptor antibodies (TRAb) and insulin‐like‐growth factor 1 (IGF‐1) acting on their receptors on fibroblasts (Girnita et al., 2022), resulting in a burst of various inflammatory mediators recruiting pluripotent bone‐marrow‐derived fibrocytes, B cells and T cells. Cell‐mediated immunity and macrophages play a key role in TED development (Avunduk et al., 2005; Chen et al., 2008; Eckstein et al., 2004; Pappa et al., 2000; Pawlowski et al., 2014). Recent studies suggest that Th17^+^ cells and their cytokines, in particular, interleukin (IL)‐13, IL‐17A and interferon (IFN)‐γ, may contribute to TED pathogenesis (Fang et al., 2019; Jiang et al., 2023).

Serum TRAb is a highly sensitive and specific biomarker for GD, but the same association is not found for TED. While nearly all patients with TED exhibit elevated TRAb levels, not all with elevated TRAb develop TED. Therefore, the diagnosis of TED relies on clinical findings (Ueland, 2023). A biomarker with high diagnostic performance is needed to guide treatment decisions to prevent progression from mild to severe TED. This is particularly important for patients scheduled for radioiodine (RAI) treatment, as it is a well‐known risk factor for de novo or flare‐ups of TED (Bartalena et al., 2021). A reliable biomarker would also be crucial in cases of TED where hyperthyroidism precedes ocular manifestations, a scenario observed in up to 30% of patients (Wiersinga et al., 1988).

As the lacrimal and Meibomian glands are frequently affected in TED (Huang et al., 2014, 2022; Kim et al., 2015; Satitpitakul et al., 2021), tear fluid is a promising source for biomarkers. Altered composition of different proteins involved in the immune system, cell cycle, metabolism as well as protein synthesis has been reported in tears from patients with TED compared to healthy subjects in proteomic studies using liquid chromatography–mass spectrometry (LC/MS) methods (Jiang et al., 2020, 2021; Kishazi et al., 2018). In addition, more conventional techniques, such as enzyme‐linked immunoassay (ELISA), have been used to validate results from LC/MS studies (Aass et al., 2017). An upregulation of inflammatory proteins and a downregulation of protective proteins have also been observed in patients with TED compared to patients with dry eye disease (Matheis et al., 2015).

Cytokines have been studied in tear fluid from TED patients due to their role in interactions between lymphocytes, macrophages and orbital fibroblasts. However, most studies are limited by small sample sizes or comparisons of TED patients only to healthy subjects (Hagan et al., 2016; Xia et al., 2006; Yang et al., 2018).

In our search for clinically useful biomarkers in TED, we analysed a panel of Th17‐related cytokines and a selection of other key candidate biomarkers in tears from GD patients with and without TED and healthy subjects. The analysis of tears was complemented by dry eye questionnaires, ocular surface evaluations and assessments of Meibomian gland function.

## 2 METHODS

### 2.1 Study subjects

A total of 106 patients with GD were recruited from routine outpatient practices at the Departments of Ophthalmology and Endocrinology, Haukeland University Hospital, Norway, between February 2022 and April 2024. The patients were selected to ensure comparable numbers in each subgroup: GD patients without TED, with active TED and with inactive TED. In addition, 106 age‐ and sex‐matched healthy subjects were recruited from hospital staff and companions of patients. Exclusion criteria for both patients and healthy subjects included the presence of other ocular diseases, use of topical medications and contact lens wear. Participants using artificial tear drops were excluded if the drops had been administered on the day of tear sampling. Informed consent was obtained from all participants. The study was approved by the Regional Committee for Medical and Health Research Ethics, Western Norway (ref. 2021/7624).

### 2.2 Clinical data

All patients had a thorough eye examination including best visual acuity, measurement of intraocular pressure and vertical palpebral fissure, Hertel's exophthalmometry and slit‐lamp biomicroscopy of the anterior part of the eye. Signs of TED including conjunctival redness, chemosis, periorbital swelling, eyelid retraction, impaired motility, strabismus and proptosis were recorded together with the following symptoms: dryness, itching, tearing, changed appearance, pain, diplopia and reduced vision.

Orbital inflammation was estimated according to the clinical activity score (CAS) (Mourits et al., 1997). Severity of TED was classified according to the European Group of Graves' Orbitopathy (EUGOGO)'s classification as mild, moderate‐to‐severe and sight‐threatening (Bartalena et al., 2021). Quality of life was assessed by a disease‐specific Graves’ Ophthalmopathy Quality of Life (GO‐QoL) questionnaire (Bartalena & Wiersinga, 2020; Terwee et al., 2001). A non‐validated Norwegian translation of the GO‐QOL questionnaire was used.

### 2.3 Definitions

GD was defined as overt thyroid dysfunction in combination with a positive TRAb. Patients were categorized as having TED if characteristic symptoms and/or signs were present. Active TED was defined as orbital inflammation with CAS ≥3. Inactive TED was defined as the absence of active orbital inflammation, corresponding to a CAS <3, and stable clinical findings for at least 6 months.

### 2.4 Collection of tear fluid

Tear fluid was collected with Schirmer test strips (Schirmer Tear Test Strips; Haag‐Streit UK, Essex, UK) without the use of topical anaesthesia. The strips were placed at the lower lateral fornix of both eyes. The participants kept their eyes closed during the procedure. After 5 min, the strips were removed with tweezers and the wetted part measured on a millimetre scale. The strips were then placed in a 1.5 mL Eppendorf Safe‐Lock micro test tube with 250 μL phosphate buffered saline and stored at −80°C until analysed.

### 2.5 Tear fluid cytokine analysis

The samples were thawed on ice, vortexed and then centrifuged at 16 000*g* for 10 min at 4°C. The protein concentration in each sample was measured using the DeNovix DS‐11 spectrophotometer (DeNovix Inc., Wilmington, DE, USA) (absorption at 280 nm). Cytokine concentrations were determined using immunoassay technology and recorded on a Luminex IS200 instrument (Bio‐Rad Laboratories, Inc., Hercules, CA, USA). Prior to the analysis, 100 μL of sample was diluted with 50 μL of calibrator diluent, RD6‐52 (R&D Systems, Abington, UK) and vortexed. Twenty‐five microlitres of each sample were assayed in duplicate. Each plate contained samples from GD patients with and without TED and healthy subjects. Wash steps were performed using the Bio‐Plex Pro wash station (Bio‐Rad Laboratories, Inc., Hercules, CA, USA). To overcome bead region incompatibilities, two custom panels were created: a 26‐plex (www.biotechne.com/l/rl/EMgbWrF4) and a 14‐plex (www.biotechne.com/l/rl/TCu994ml). An in‐house control material was used to determine inter and intra assay variation. Only participants with successful protein determination of the tear samples were included in the study.

The following 23 biomarkers related to Th17 response were analysed: CD40 Ligand (CD40L), chemokine (C–C motif) ligand 20 (CCL20), granulocyte‐macrophage colony‐stimulating factor (GM‐CSF), IFN‐©, IL‐1β, IL‐2, IL‐4, IL‐5, IL‐6, IL‐10, IL‐12p70, IL‐13, IL‐15, IL‐17A, IL‐17E, IL‐21, IL‐23, IL‐27, IL‐28A, IL‐31, IL‐33, lymphotoxin‐α (LT‐α) and tumour necrosis factor (TNF).

In addition, 17 analytes were analysed as potential biomarkers based on previous studies (Gonnella, 2019; Han et al., 2021; Huang et al., 2012; Matheis et al., 2015; Song et al., 2020; Ueland et al., 2022; Vannucchi et al., 2012); B‐cell activating factor (BAFF), chemokine (C–C motif) ligand 2 (CCL2), chemokine (C–C motif) ligand 7 (CCL7), chemokine (C–X–C motif) ligand 9 (CXCL9), chemokine (C–X–C motif) ligand 10 (CXCL10), chemokine (C–X–C motif) ligand 11 (CXCL11), Chitinase 3‐like 1 (CHI3L1), C‐reactive protein (CRP), FMS‐like tyrosine kinase 3 ligand (Flt3L), IFN‐α, IL‐8, macrophage colony‐stimulating factor (M‐CSF), platelet‐derived growth factor (PDGF‐AA), S100 calcium‐binding protein A8 (S100A8), TNF receptor superfamily member 9 (TNFRSF9), vascular cell adhesion protein 1 (VCAM‐1) and vitamin D binding protein (VitDBP).

### 2.6 Ocular surface examination

All participants completed the validated Ocular Surface Disease Index (OSDI) questionnaire to assess for symptoms of dry eye disease (Schiffman et al., 2000). The subjective score ranges from 0 to 100 and higher scores reflect more discomfort and impact on the patient's quality of life. In addition, tear film fluorescein break‐up time (TBUT) was measured and keratopathy was evaluated according to the Oxford grading scheme (Bron et al., 2003).

Meibomian gland function was assessed by both meibum expressibility (ME) and meibum quality (MQ), with higher scores indicating reduced function. ME was graded by the number of the central glands in the lower eyelid that demonstrated secretion (0–3 points). The recommendations from the International Workshop on Meibomian gland dysfunction were used to grade MQ (0–24 points) (Tomlinson et al., 2011).

### 2.7 Statistical analysis

Data analysis was performed using SPSS Version 29.0 and R version 4.2.3 (R Core Team, 2023). Descriptive statistics characterised GD patients and healthy subjects, reporting continuous data as median (min–max) and categorical data as counts (percentage). Mann–Whitney *U* test was used for comparison of continuous variables across groups, whereas Pearson's chi‐squared or Fisher's exact test, depending on expected counts, was used for comparison of categorical variables across groups. Spearman's rank correlation assessed correlations between continuous and ordinal variables, and point‐biserial correlation between continuous and binary variables (sex, smoking). A correlation coefficient was considered important if *p* < 0.05 and *r* > 0.4.

Differences in biomarker levels between disease groups were investigated using linear regression analysis, with the biomarker being the continuous dependent variable and the disease group being the binary independent variable. To address right‐skewed distributions, all biomarkers were log‐transformed before analyses using the natural logarithm scale. All analyses were adjusted for sex, age, BMI, smoking and the Oxford grading score, and the result for each biomarker was computed as the mean difference of log‐transformed values with the corresponding 95% confidence interval (CI) and p‐value. Both the 95% CI and *p*‐value were calculated based on cluster robust standard errors due to the matching of cases and controls.

To ensure a complete sample for the regression analyses, missing data in the adjustment variables were imputed once using the predictive mean matching algorithm (Van Buuren & Groothuis‐Oudshoorn, 2011). For biomarker values below detection limits, random values were imputed between the lower limit of detection (LLOD) and half of this minimum, thus maintaining variability without losing information. The level of statistical significance was set to 0.01, corresponding to a –log 10 (*p‐*value) of 2. Differences in biomarker values between the two groups were presented by Volcano plots.

Receiver operating characteristic (ROC) analysis was used to evaluate biomarker performance. A fixed specificity of 0.80 was used to emphasise diagnostic performance in a clinically relevant setting where false positives are limited to 20%.

## 3 RESULTS

### 3.1 Study population

In total, 106 GD patients (82 females) with a median (min–max) age of 48.5 (20–81) years were included in the study (Table 1). Median BMI was 25.3 (18.4–63.7) kg/m^2^. The number of smokers was significantly higher among the GD patients (13 out of 106) compared to the healthy subjects (2 out of 106) (*p* = 0.009).

**TABLE 1:** Basic characteristics, blood levels, treatment of hyperthyroidism and ocular surface parameters at inclusion.

Parameter	Patients with GD			Healthy subjects	*p* values*
	All	Without TED	With TED		
Basic characteristics					
Patients, *n*	106	36	70	106	
Age, years (min–max)	48.5 (20–81)	45 (23–75)	51 (20–81)	48.5 (20–81)	1/0.096
Female, *n* (%)	82 (77.4)	28 (77.8)	54 (77.1)	82 (77.4)	1/0.941
Daily smoker, *n* (%)	13 (12.3)	4 (11.1)	9 (12.9)	3 (2.8)	0.009/1
Body mass index, kg/m^2^ (min–max)	25.30 (18.4–63.7)	27.3 (19.3–37.2)	25.2 (18.4–63.7)	25.0 (17.6–35.6)	0.340/0.043
Laboratory tests					
s‐free‐Thyroxine, pmol/L (min–max)	17.4 (8.4–59.0)	18.4 (11.6–59.0)	17.4 (8.4–42.5)		0.348
s‐Triiodothyronine, pmol/L (min–max)	5.2 (2.4–22.0)	5.9 (3.4–22.0)	4.8 (2.4–19.8)		0.003
TRAb, IU/L (min–max)	4.5 (1.0–40.0)	3.7 (1.0–30.3)	5.0 (1.0–40.0)		0.997
TPO‐ab >200 kIU/L, *n* (%)	13 (16.7)	6 (21.4)	7 (14.0)		0.528
Type of GD treatment					
Titration with carbimazole, *n* (%)	46 (43.4)	22 (61.1)	24 (34.3)		0.008
Titration with propylthiouracil, *n* (%)	4 (3.8)	1 (2.8)	3 (4.3)		1
Blocking with carbimazole, *n* (%)	16 (15.1)	1 (2.8)	15 (21.4)		0.011
Blocking with propylthiouracil, *n* (%)	1 (0.9)	0 (0)	1 (1.4)		1
Levaxin substitution after thyroidectomy, *n* (%)	20 (18.9)	2 (5.6)	18 (25.7)		0.012
No treatment of hyperthyroidism, *n* (%)	18 (17.0)	10 (27.8)	8 (11.4)		0.034
Unknown treatment of hyperthyroidism, *n* (%)	1 (0.9)	0 (0)	1 (1.4)		1
Ocular surface parameters					
Schirmer's test, mm (min–max)	18 (0–40)	15.5 (0–40)	20 (0–40)	16 (0–40)	0.70/0.440
Fluorescein break‐up time, seconds (min–max)	7 (2–31)	7 (3–25)	6.50 (2–31)	9 (2–26)	0.351/0.099
Ocular Surface Disease Index score (min–max)	21.60 (0–86.40)	7.29 (0–45.80)	31.38 (0–86.40)	2 (0–81.81)	<0.01/<0.01
Oxford grading score (min–max)	0 (0–3)	0 (0–3)	0.50 (0–3)	0 (0–3)	0.114/0.65
Meibum quality (min–max)	0 (0–16)	0 (0–8)	0 (0–16)	0 (0–8)	0.238/0.317
Meibum expressibility (min–max)	0 (0–3)	0 (0–3)	0 (0–2)	0 (0–3)	0.648/0.093

*Note*: Categorical data are presented as *n* (%); continuous data are presented as median (min–max).

Abbreviations: GD, Graves’ disease; TED, thyroid eye disease; TPOab, thyroid peroxidase antibody; TRAb, thyrotropin receptor antibody; TSH, thyroid stimulating hormone.

* *p*‐values: ‘Basic characteristics’ and ‘ocular surface parameters’: between GD and healthy subjects/between GD with and without TED. ‘Laboratory levels’ and ‘type of treatment’: between GD with and without TED.

Seventy (66.0%) patients had TED at inclusion, of which 32 (30.2%) had active orbital inflammation. Fifty (47.2%) patients had moderate‐to‐severe or sight‐threatening TED (Table 2). Patients with TED had significantly more proptosis (18 (12–25) mm) compared to those without TED (16 (10–22) mm) (*p* < 0.01). Similarly, the vertical eyelid aperture was larger in TED patients (10 (7–17) mm) than in GD patients without TED (10 (7–14) mm) (*p* = 0.009).

**TABLE 2:** Clinical characteristics and treatment of 70 patients with TED at inclusion.

Parameter	No. (%)
Clinical characteristics	
CAS ≥3 at inclusion	32 (45.7)
EUGOGO classification >1	50 (71.4)
Changed appearance	57 (81.4)
Pain/pressure	30 (42.9)
Diplopia	19 (9.0)
Reduced vision	12 (5.7)
Red eye	26 (37.1)
Chemosis	19 (27.1)
Periorbital swelling	43 (48.6)
Eyelid retraction	36 (51.4)
Strabismus	13 (18.6)
Proptosis	30 (42.9)
Treatment	
Corticosteroids	24 (34.3)
Corticosteroids + rituximab	1 (1.4)
Corticosteroids + irradiation	3 (4.3)
Orbital decompression	10 (14.3)
Strabismus surgery	2 (2.9)
Eyelid surgery	6 (8.6)
Orbital decompression + eyelid surgery	4 (4.3)
Orbital decompression + strabismus surgery + eyelid surgery	4 (5.7)

Abbreviations: CAS, clinical activity score; EUGOGO, European Group of Graves' orbitopathy; TED, thyroid eye disease.

### 3.2 Biomarkers

**FIGURE 1:**
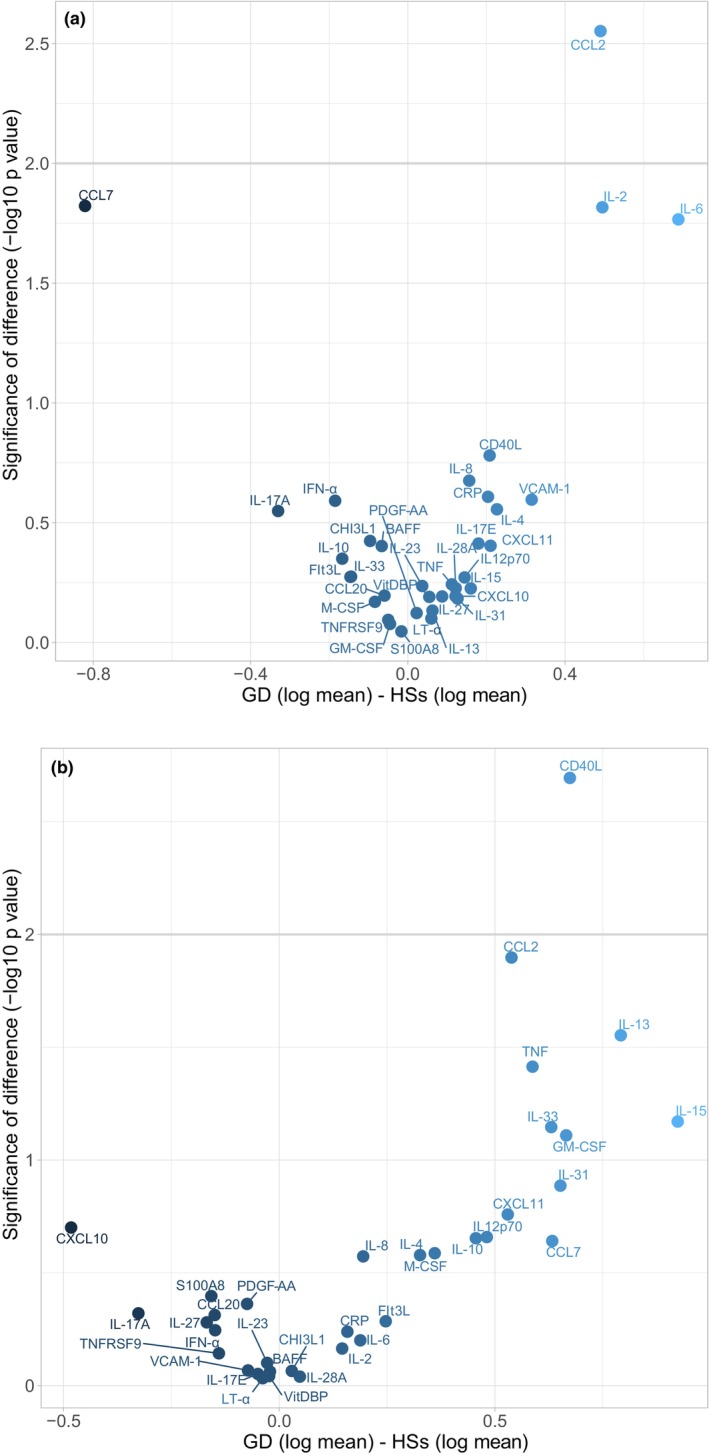
(a) Volcano plot comparing 35 inflammatory biomarkers in tear fluid from 106 patients with Graves' disease (GD) and 106 healthy subjects (HSs). BAFF, B‐cell activating factor; CCL2, chemokine (C‐C motif) ligand 2; CCL7, chemokine (C‐C motif) ligand 7; CCL20, chemokine (C‐C motif) ligand 20; CD40L, CD40 ligand; CHI3L, chitinase 3‐like 1; CRP, C‐reactive protein; CXCL10, chemokine (C‐X‐C motif) ligand 10; CXCL11, chemokine (C‐X‐C motif) ligand 11; Flt3L, FMS‐like tyrosine kinase 3 ligand; GM‐CSF, granulocyte‐macrophage colony‐stimulating factor; IFN‐α, interferon α; IL‐2, interleukin 2; IL‐4, interleukin 4; IL‐6, interleukin 6; IL‐8, interleukin 8; IL‐10, interleukin 10; IL‐12p70, interleukin 12p70; IL‐13, interleukin 13; IL‐15, interleukin 15; IL‐17A, interleukin 17A; IL‐17E, interleukin 17 E; IL‐23, interleukin 23; IL‐27, interleukin 27; IL‐28A, interleukin 28A; IL‐31, interleukin 31; IL‐33, interleukin 33; LT‐α, lymphotoxin‐α; M‐CSF, macrophage colony‐stimulating factor; PDGF‐AA, platelet derived growth factor; S100A8, S100 calcium‐binding protein A8; TNF, tumour necrosis factor; TNFRSF9, TNF receptor superfamily member 9; VitDBP, vitamin D binding protein; VCAM‐1, vascular cell adhesion protein 1. (b) Volcano plot comparing 35 inflammatory biomarkers in tear fluid from Graves' disease patients with thyroid eye disease (TED, *n* = 70) and without (noTED, *n* = 36). BAFF, B‐cell activating factor; CCL2, chemokine (C–C motif) ligand 2; CCL7, chemokine (C–C motif) ligand 7; CCL20, chemokine (C–C motif) ligand 20; CD40L, CD40 ligand; CHI3L, chitinase 3‐like 1; CRP, C‐reactive protein; CXCL10, chemokine (C–X–C motif) ligand 10; CXCL11, chemokine (C–X–C motif) ligand 11; Flt3L, FMS‐like tyrosine kinase 3 ligand; GM‐CSF, granulocyte‐macrophage colony‐stimulating factor; IFN‐α, interferon α; IL‐2, interleukin 2; IL‐4, interleukin 4; IL‐6, interleukin 6; IL‐8, interleukin 8; IL‐10, interleukin 10; IL‐12p70, interleukin 12p70; IL‐13, interleukin 13; IL‐15, interleukin 15; IL‐17A, interleukin 17A; IL‐17E, interleukin 17E; IL‐23, interleukin 23; IL‐27, interleukin 27; IL‐28A, interleukin 28A; IL‐31, interleukin 31; IL‐33, interleukin 33; LT‐α, lymphotoxin‐α; M‐CSF, macrophage colony‐stimulating factor; PDGF‐AA, platelet‐derived growth factor; S100A8, S100 calcium‐binding protein A8; TNF, tumour necrosis factor; TNFRSF9, TNF receptor superfamily member 9; VitDBP, vitamin D binding protein; VCAM‐1, vascular cell adhesion protein 1.

The following five biomarkers were excluded from analysis due to a high proportion (>50%) of values below the lower limit of detection (LLOD): CXCL9, IL‐5, IL‐21, IL‐1β and IFN‐γ. Of the remaining 35 biomarkers (Table 3, Table S1), tear fluid levels of CCL2 were significantly higher in GD patients compared to healthy subjects (*p* = 0.003) (Figure 1). GD patients also had higher levels of IL‐2 and IL‐6 (*p* = 0.015 and *p* = 0.017, respectively), but lower levels of CCL7 (*p* = 0.015) compared to healthy subjects. The p‐values for these differences were close to the predefined significance level of 0.01.

**TABLE 3:** Levels of inflammatory tear fluid biomarkers in patients with Graves’ disease and healthy subjects.

Biomarker^a^	Patients with GD			Healthy subjects	LLOD	CV	Adjusted *p*‐values
	All	Without TED	With TED				
	106	36	70	106			
Th‐17 response							
CD40 Ligand (CD40L)	350.9 (267.6)	300.6 (290.2)	376.7 (253.5)	336.8 (274.3)	5.4	5.6	0.166/0.002
Chemokine (C‐C motif) ligand 20 (CCL20)	93.0 (119.7)	91.1 (69.5)	94.0 (139.2)	90.8 (72.0)	0.2	11.5	0.637/0.487
Granulocyte‐macrophage colony‐stimulating factor (GM‐CSF)	36.6 (58.1)	32.9 (41.5)	38.5 (65.2)	39.3 (57.9)	0.4	9.9	0.837/0.078
Interleukin‐2 (IL‐2)	11.0 (13.0)	9.3 (9.6)	11.9 (14.4)	6.3 (7.4)	0.8	5.2	0.015/0.784
Interleukin‐4 (IL‐4)	23.9 (22.6)	20.7 (21.5)	25.5 (23.1)	24.1 (32.9)	1.8	5.0	0.278/0.264
Interleukin‐6 (IL‐6)	3.3 (5.2)	2.4 (3.1)	3.8 (5.9)	2.2 (3.4)	0.04	2.4	0.017/0.630
Interleukin‐10 (IL‐10)	14.4 (19.6)	13.9 (19.1)	14.6 (20.0)	16.0 (21.1)	0.6	18.3	0.447/0.222
Interleukin‐12p70 (IL‐12p70)	229.5 (437.6)	144.7 (157.6)	273.1 (522.6)	186.1 (250.0)	7.9	6.3	0.535/0.219
Interleukin‐13 (IL‐13)	96.5 (150.8)	77.4 (137.0)	106.3 (157.4)	108.3 (188.3)	8.6	4.3	0.736/0.028
Interleukin‐15 (IL‐15)	3.4 (4.0)	2.0 (3.0)	4.1 (4.2)	2.9 (3.5)	0.1	4.5	0.595/0.068
Interleukin‐17A (IL‐17A)	8.6 (13.1)	10.0 (14.3)	7.9 (12.6)	10.7 (15.6)	0.2	4.6	0.283/0.478
Interleukin‐17 E (IL‐17E)	190.6 (158.6)	171.1 (129.8)	200.7 (171.5)	187.7 (201.5)	3.4	11.9	0.386/0.888
Interleukin‐23 (IL‐23)	811.7 (388.2)	798.6 (382.3)	818.4 (393.7)	807.6 (497.7)	53.6	1.4	0.581/0.793
Interleukin‐27 (IL‐27)	70.4 (96.3)	64.7 (47.7)	73.4 (113.7)	55.9 (45.9)	2.2	3.0	0.642/0.524
Interleukin‐28A (IL‐28A)	31.8 (60.1)	25.2 (30.6)	35.3 (70.6)	23.6 (29.3)	1.0	1.9	0.593/0.912
Interleukin‐31 (IL‐31)	68.3 (74.4)	59.6 (74.5)	72.8 (74.4)	66.5 (89.5)	1.6	5.0	0.656/0.130
Interleukin‐33 (IL‐33)	7.8 (13.4)	6.7 (13.1)	8.3 (13.7)	7.6 (9.3)	0.6	4.4	0.531/0.071
Lymphotoxin‐α (LT‐α)	8.3 (8.4)	6.9 (7.0)	9.1 (9.1)	8.8 (11.2)	0.1	11.2	0.794/0.927
Tumour necrosis factor (TNF)	5.1 (6.0)	3.5 (3.9)	5.9 (6.7)	4.5 (5.5)	0.3	4.4	0.572/0.039
Other biomarkers							
B‐cell activating factor (BAFF)	27.2 (14.6)	26.4 (10.8)	27.6 (16.2)	28.5 (13.9)	4.9	4.2	0.396/0.866
Chemokine (C–C motif) ligand 2 (CCL2)	89.0 (192.9)	53.4 (53.4)	107.3 (232.7)	58.4 (92.6)	5.3	6.5	0.003/0.013
Chemokine (C–C motif) ligand 7 (CCL7)	53.6 (127.7)	44.1 (135.7)	58.4 (124.1)	83.8 (201.1)	0.7	7.2	0.015/0.228
Chemokine (C–X–C motif) ligand 10 (CXCL10)	17800.8 (53786.8)	8577.0 (13358.7)	22544.4 (65151.3)	20631.3 (62431.8)	0.6	2.7	0.641/0.199
Chemokine (C–X–C motif) ligand 11 (CXCL11)	40.9 (45.2)	33.2 (42.1)	44.9 (46.6)	32.1 (33.1)	0.9	6.8	0.395/0.174
Chitinase 3‐like 1 (CHI3L)	4285.1 (4068.0)	4104.5 (4124.9)	4378.0 (4065.2)	4542.7 (3446.8)	131.9	3.6	0.377/0.861
C‐reactive protein (CRP)	1300.9 (1870.9)	1611.5 (2136.3)	1141.1 (1713.2)	1054.1 (1845.7)	29.6	9.8	0.246/0.577
FMS‐like tyrosine kinase 3 ligand (Flt3L)	11.4 (9.7)	11.1 (10.5)	11.7 (9.4)	12.7 (11.9)	0.1	5.2	0.532/0.518
Interferon‐α (IFN‐α)	19.1 (20.0)	18.7 (20.8)	19.3 (19.7)	22.6 (28.1)	0.6	10.7	0.256/0.568
Interleukin‐8 (IL‐8)	107.1 (103.7)	89.8 (78.4)	116.0 (114.0)	97.7 (106.4)	2.2	5.4	0.211/0.267
Macrophage colony‐stimulating factor (M‐CSF)	125.6 (148.7)	105.9 (155.1)	135.8 (145.4)	146.7 (214.4)	1.9	4.6	0.676/0.259
Platelet‐derived growth factor (PDGF‐AA)	93.2 (41.8)	100.4 (44.2)	89.4 (40.4)	94.5 (47.0)	1.3	11.3	0.754/0.434
S100 calcium‐binding protein A8 (S100A8)	404.3 (470.2)	462.2 (488.0)	374.6 (461.5)	411.3 (526.8)	19.1	4.9	0.899/0.401
TNF receptor superfamily member 9 (TNFRSF9)	6.2 (9.7)	6.3 (8.1)	6.1 (10.5)	7.0 (10.9)	0.4	5.9	0.805/0.719
Vascular cell adhesion protein 1 (VCAM‐1)	2897.7 (3010.3)	2489.2 (2823.5)	3107.8 (3100.6)	2558.2 (3019.1)	38.6	3.1	0.253/0.856
Vitamin D binding protein (VitDBP)	22041.2 (17416.0)	22513.7 (18394.9)	21798.2 (17022.2)	20946.7 (16510.9)	1897.6	13.4	0.645/0.907

*Note*: Data presented as the mean (SD). *p* values: comparing GD and healthy subjects, in addition to GD with and without TED. *p*‐values for group differences in biomarkers were calculated based on log‐transformed values and were adjusted for age, sex, smoking, BMI and the Oxford grading score.

Abbreviations: CV, coefficient of variation; GD, Graves' disease; LLOD, lower limit of detection.

^a^ Pg per mL.

When comparing tear fluid levels in GD patients with and without TED, CD40L was significantly higher in the patients with TED (*p* = 0.002) (Figure 1b). A similar group difference was also observed for CCL2 (*p* = 0.013). No significant differences in any other biomarker were observed between TED patients with active and inactive disease or between those with moderate‐to‐severe or sight‐threatening TED and those with mild TED.

We did not observe important correlations (*p* < 0.05, *r* > 0.4) between any of the two candidate biomarkers (CD40L and CCL2), and serum levels of TRAb and fT4, and various basic characteristics such as age, sex, BMI and smoking status as well as ocular surface variables including Schirmer's test, TBUT, MQ, ME, Oxford grading score and OSDI.

### 3.3 Ocular surface evaluation

GD patients had significantly higher OSDI scores compared to healthy subjects, with median scores of 21.6 (0–86.4) and 2.0 (0–81.8), respectively (*p* < 0.01) (Table 1). Further, GD patients with TED had significantly higher OSDI scores (31.4 (0–86.4)) compared to GD patients without TED (7.3 (0–45.8)) (*p* < 0.01). Both subgroups of patients with TED and patients without TED had significantly higher OSDI scores compared to their matched healthy subjects.

We observed that 11 of 50 patients with moderate‐to‐severe or sight‐threatening TED had an MQ score above seven compared to 0 of 20 patients with mild TED (*p* = 0.027). No significant differences were observed in MQ score between GD patients with and without TED or between GD patients and healthy subjects.

In addition, no significant differences in ME, TBUT, tear production (Schirmer's test) and Oxford grading score were observed between the following groups: GD patients and healthy subjects, GD patients with and without TED, patients with active and inactive TED and TED patients with moderate‐to‐severe or sight‐threatening compared to mild disease.

Among TED patients, no important correlations were observed between the degree of proptosis and Schirmer's test, TBUT, Oxford grading score or OSDI. Similarly, vertical eyelid aperture showed no correlation with any of the ocular surface parameters.

### 3.4 Diagnostic performance

**FIGURE 2:**
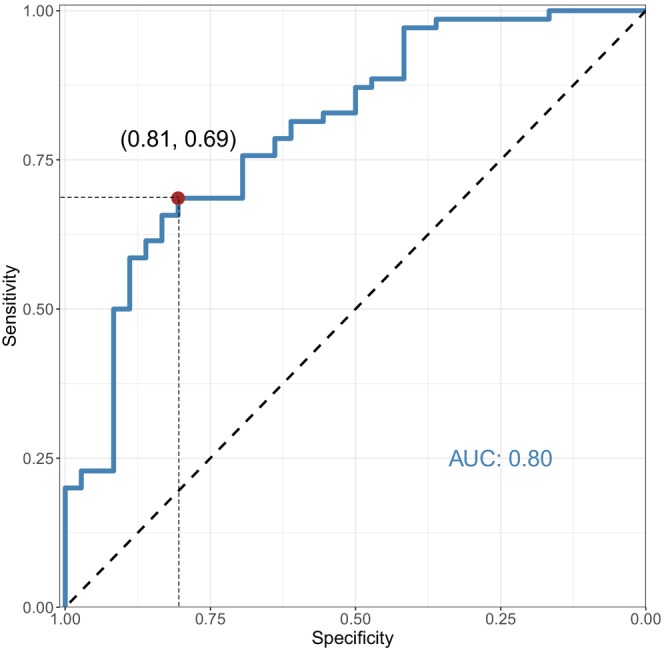
Receiver Operating Characteristic curve, with Ocular Surface Disease Index and tear fluid levels of CD40 Ligand and chemokine (C–C motif) ligand 2 (CCL2) combined, demonstrating an area under the curve (AUC) of 0.80, with sensitivity 0.69 at a fixed specificity of 0.80. The true positive rate (sensitivity) is plotted against the true negative rate (specificity).

To discriminate GD patients with TED from those without, we found the OSDI score to have an area under the ROC curve of 0.76 with a corresponding sensitivity of 0.61 at a fixed specificity of 0.80. For the same diagnostic purpose, the tear fluid levels of CCL2 yielded an area under the ROC curve of 0.66 with a sensitivity of 0.34 at a specificity of 0.80. Further, we found an area under the ROC curve of 0.63 with a sensitivity of 0.23 at a specificity of 0.80 for CD40L. By combining the OSDI score with the tear fluid levels of CCL2 and CD40L, the area under the ROC curve improved to 0.80 with sensitivity of 0.69 at a fixed specificity of 0.80 (Figure 2).

## 4 DISCUSSION

We found elevated tear fluid levels of CD40L and CCL2 in GD patients with TED compared to those without. Furthermore, GD patients with TED reported more dry eye symptoms, as measured by the OSDI score than GD patients without TED, who in turn had more symptoms than healthy subjects. Patients with moderate‐to‐severe and sight‐threatening TED had worse MQ compared to patients with mild TED. These findings indicate that tear fluid levels of CD40L and CCL2 may reflect the pathological processes in TED and represent potential biomarkers, together with assessment of ocular surface parameters.

Of 23 biomarkers related to the Th17 response, only CD40L levels were increased in tear fluid in patients with TED compared to GD patients without, a finding not previously reported. CD40 and CD40L have earlier only been found to be higher in patients with TED compared to healthy subjects (Song et al., 2020). Our finding is consistent with studies that link CD40L to the pathogenesis of TED. Orbital fibroblasts from TED patients have been observed to express the co‐stimulatory protein CD40 (Grewal & Flavell, 1996). Binding of orbital fibroblasts to CD40L on T cells is proposed to activate the fibroblasts to release immune mediators including IL‐6, IL‐8 and CCL2 (Feldon et al., 2005; Hwang et al., 2009; Sempowski et al., 1998). These findings are in line with earlier observations of higher serum levels of CD40 in GD patients with relapsing or persistent disease compared to patients who maintained normal thyroid function after anti‐thyroid drug treatment (Meling Stokland et al., 2024). Interestingly, treatment with Iscalimab, a CD40 inhibitor, in a small proof‐of‐concept study, improved orbitopathy in two out of six patients following normalization of thyroid function (Kahaly et al., 2020). Our findings of increased tear fluid levels of CD40L in TED support further investigation of anti‐CD40 treatment in TED.

We also found increased tear fluid levels of CCL2 in GD patients with TED compared to those without TED. This is in coherence with a previous report of higher tear fluid levels of CCL2 in GD patients without TED compared to healthy subjects (Mandić et al., 2018). The main mechanism of CCL2 is to stimulate monocytes to migrate from the circulation, infiltrate and develop into residential macrophages, thereby playing a vital role in inflammation. In orbital fat, high expression of CCL2, along with infiltration of macrophages, has been observed in TED patients (Chen et al., 2008). Several studies have reported increased secretion of CCL2 from orbital fibroblasts after stimulation with proinflammatory cytokines in TED (Elner et al., 1998; Han et al., 2021; Unsworth et al., 2020). These findings, along with our observations, support the crucial role of CCL2 in the pathogenesis of TED.

We found higher OSDI scores in GD patients compared with healthy subjects. GD patients with TED also reported more dry eye symptoms than GD patients without TED. As GD patients with TED had more proptosis and a larger vertical eyelid aperture compared to those without TED, one may assume that these conditions cause increased evaporation, subjective dry eye symptoms, and signs of ocular surface changes. However, no correlations were found between the clinical parameters (degree of proptosis and vertical eyelid aperture) and Schirmer's test, TBUT, Oxford grading score or OSDI. In addition, in line with previous studies, we did not find significant changes in any of these dry eye‐related variables between GD patients with and without TED (Gürdal et al., 2011; Kashkouli et al., 2018). EUGOGO recommends extensive treatment with artificial tears in all patients with TED (Bartalena et al., 2021). Our study, along with previous studies, indicates that ocular surface inflammation could be present in patients with GD, even in the absence of overt TED. We therefore support the liberal use of artificial tears in any GD patients with ocular discomfort.

In line with a recent study by Aghaei et al., we observed that patients with moderate‐to‐severe and sight‐threatening TED had worse MQ scores than those with mild disease (Aghaei et al., 2024). This is supported further by another study indicating that mechanical factors contribute to dry eye disease in both active and chronic TED, with Meibomian gland dysfunction only being significant during the active phase (Allam et al., 2021). Similarly, a previous study found significant loss of Meibomian glands in TED patients, which positively correlated with CAS (Kim et al., 2015). Their TED patients also had higher OSDI scores and lower TBUT. A study from Satitpitakul and co‐authors showed that patients with inactive TED had significant Meibomian gland loss compared to healthy subjects, but no significant differences in OSDI, corneal staining or TBUT (Satitpitakul et al., 2021). Interestingly, their TED patients had better MQ and ME scores than healthy subjects. The somewhat conflicting results from previous studies regarding ocular surface symptoms and Meibomian glands highlight the complex inflammatory involvement in TED and underscore that a functional assessment of Meibomian glands should be incorporated in the evaluation of patients with TED, especially in more severe disease.

Identifying patients with subclinical TED is crucial, as it could reduce the risk of exacerbating TED through radioiodine treatment (Vannucchi et al., 2009). We found that the OSDI score, as well as the tear fluid levels of CCL2 and CD40L, each had only moderate diagnostic performance to identify TED patients. However, when combined, these biomarkers showed an area under the ROC curve of 0.80. Proteomic profiling of tear fluid proteins has shown better performance in discriminating between patients with TED and healthy subjects (Okrojek et al., 2009) and between GD patients with and without TED (Aass et al., 2017). The later study introduced a panel of tear fluid biomarkers consisting of lysozyme C, lacritin, and zinc‐α‐2 glycoprotein, which differentiated between GD patients with and without TED, with an area under the ROC curve of 0.93 (Aass et al., 2017). However, our combined biomarker is easier to perform and may be less influenced by confounding factors, as CD40L and CCL2 are likely more specifically associated with the pathogenesis of TED.

A strength of our study is the high sensitivity of the multiplex bead assay in tear fluid analysis (Huang et al., 2012; Landsend et al., 2018; Xu et al., 2020). Luminex enables the simultaneous testing of a panel of biomarkers, and it provides a straightforward approach by reducing the complexity of potential irrelevant findings and matrix effects often seen using LC–MS. Also, our study encompassed GD patients both with and without TED, offering a more comprehensive approach compared to most existing studies on tear–fluid biomarkers, which predominantly focus on comparing only TED patients with healthy subjects. A limitation of the study is that the patients included do not represent randomly selected GD patients. To have approximately the same number of patients in each category (GD without TED, GD with active TED and GD with inactive TED), the proportion of patients with TED is higher and the eye disease is more severe than would be expected in a randomly selected group of GD patients (Chin et al., 2020). Biomarkers primarily associated with TED, in particular with active disease, could therefore become more strongly associated with GD than would be the case in randomly selected GD patients. Also, tear fluid samples were obtained strictly from the patient's right eye to avoid selection bias. Although bilateral presentation is most common, TED may present asymmetrically (Eshraghi et al., 2023). In addition, we evaluated Meibomian gland function using established subjective measures and clinical observations. The inclusion of meibography, however, could provide a more objective and comprehensive assessment.

In conclusion, our study reveals elevated CD40L and CCL2 levels in tear fluid from GD patients with TED, indicating their role as key pathogenic mediators. Combined with OSDI, they showed promising diagnostic performance, and we recommend further evaluation of these three parameters in the diagnostic workup for TED. Additionally, given the observed ocular discomfort and reduced meibomian gland quality in TED patients, we suggest liberal use of artificial tears and routine assessment of meibomian gland function, especially in moderate to severe cases.

## FUNDING INFORMATION

This work was supported by grants from the Western Norway Regional Health Authority and the Novo Nordisk Foundation.

## CONFLICT OF INTEREST STATEMENT

None of the authors has any commercial or financial associations that might pose or create a conflict of interest with information presented in this article.

## Supporting information


Table S1.
