# Supplementary material for: Exploring tear fluid biomarkers and the ocular surface in thyroid eye disease
Source: Acta Ophthalmol. 2025 Jul 7;104(1):e28–38. doi: 10.1111/aos.17556 (PMC12803598; doi:10.1111/aos.17556)
Supplement: Supplementary file 1 — Table S1. [file AOS-104-e28-s001.docx]

|  | | | | |  |
| --- | --- | --- | --- | --- | --- |
|  | | | | |  |
| **Supplemental Table.** Levels of inflammatory tear fluid biomarkers in patients with Graves` disease and healthy subjects | | | | |  |
| **Biomarker^*^** |  | **Patients with GD** |  | **Healthy subjects** |  |
|  | **All** | **Without TED** | **With TED** |  |  |
|  | 106 | 36 | 70 | 106 | |
| **Th-17 response** |  |  |  |  | |
| [CD40 Ligand](https://www.rndsystems.com/products/human-luminex-discovery-assay_lxsahm) (CD40L) | 306.4 (4.6-1323.6) | 203.9 (4.6-1294.6) | 319.8 (59.3-1323.6) | 270.4 (2.8-1559.6) | |
| Chemokine (C-C motif) ligand 20 (CCL20) | 60.6 (0.2-1102.8) | 75.2 (4.9-323.8) | 56.0 (0.2-1102.8) | 69.6 (0.2-474.5) | |
| Granulocyte-macrophage colony-stimulating factor (GM-CSF) | 19.7 (0.3-433.6) | 21.6 (0.3-200.2) | 19.6 (0.3-433.6) | 20.6 (0.2-364.6) | |
| Interleukin 2 (IL-2) | 6.9 (0.4-62.9) | 5.6 (0.4-31.7) | 7.1 (0.4-62.9) | 3.3 (0.4-34.2) | |
| Interleukin 4 ([IL-4](https://www.rndsystems.com/products/human-luminex-discovery-assay_lxsahm)) | 16.3 (0.9-123.0) | 11.2 (0.9-83.1) | 19.6 (1.0-123.0) | 12.9 (0.9-196.2) | |
| Interleukin 6 (IL-6) | 2.0 (0.0-32.9) | 1.3 (0.0-17.0) | 2.1 (0.0-32.9) | 1.0 (0.0-25.0) | |
| Interleukin 10 (IL-10) | 9.7 (0.3-141.4) | 8.4 (0.3-92.6) | 9.9 (0.3-141.4) | 9.1 (0.3-90.7) | |
| Interleukin 12p70 (IL-12p70) | 108.0 (4.0-3921.3) | 81.1 (4.0-600.9) | 111.0 (4.1-3921.3) | 117.0 (4.0-1318.2) | |
| Interleukin 13 (IL-13) | 40.4 (4.3-967.3) | 7.6 (4.3-584.4)^†^ | 72.0 (4.3-967.3) | 44.2 (4.4-1522.2) | |
| Interleukin 15 (IL-15) | 2.1 (0.0-20.3) | 0.0 (0.0-14.3)^†^ | 3.4 (0.0-20.3) | 1.6 (0.0-16.8) | |
| Interleukin 17A (IL-17A) | 3.6 (0.1-70.9) | 3.8 (0.1-52.0) | 3.6 (0.1-70.9) | 4.6 (0.1-78.6) | |
| Interleukin 17 E (IL-17E) | 176.3 (1.8-1012.5) | 146.5 (2.0-532.8) | 185.9 (1.8-1012.5) | 153.9 (1.8-1749.9) | |
| Interleukin 23 (IL-23) | 760.4 (207.2-2005.8) | 715.5 (232.5-1937.7) | 787.7 (207.2-2005.8) | 689.2 (27.1-3641.3) | |
| Interleukin 27 (IL-27) | 54.5 (1.3-906.5) | 56.1 (1.7-243.4) | 53.9 (1.3-906.5) | 42.3 (1.2-250.4) | |
| Interleukin 28A (IL-28A) | 13.9 (0.5-343.4) | 17.9 (0.6-125.7) | 13.2 (0.5-343.4) | 14.6 (0.5-176.1) | |
| Interleukin 31 (IL-31) | 51.3 (0.8-375.1) | 25.5 (0.9-311.9) | 58.8 (0.8-375.1) | 46.1 (0.9-589.9) | |
| Interleukin 33 (IL-33) | 1.8 (0.3-71.2) | 0.6 (0.3-52.6) | 4.3 (0.3-71.2) | 4.4 (0.3-52.4) | |
| [Lymphotoxin-α (LT-α)](https://www.rndsystems.com/products/human-luminex-discovery-assay_lxsahm) | 5.7 (0.0-36.7) | 5.5 (0.1-36.7) | 6.1 (0.0-35.2) | 5.5 (0.0-79.1) | |
| Tumor necrosis factor (TNF) | 3.6 (0.2-47.4) | 3.0 (0.2-18.7) | 4.0 (0.2-47.4) | 3.3 (0.2-40.7) | |
| **Other biomarkers** |  |  |  |  | |
| B-cell activating factor (BAFF) | 25.2 (4.6-76.8) | 26.6 (4.6-44.7) | 23.3 (4.9-76.8) | 25.9 (2.8-69.2) | |
| Chemokine (C-C motif) ligand 2 (CCL2) | 51.6 (4.6-1923.2) | 34.5 (4.6-199.1) | 60.9 (4.9-1923.2) | 28.5 (3.0-710.1) | |
| Chemokine (C-C motif) ligand 7 (CCL7) | 8.3 (0.3-815.5) | 0.6 (0.3-758.1)^†^ | 13.9 (0.3-815.5) | 28.7 (0.3-1454.1) | |
| Chemokine (C-X-C motif) ligand 10 (CXCL10) | 2510.5 (151.1-245846.0) | 2874.3 (151.1-37707.4) | 2510.5 (154.2-245846.0 | 2204.5 (0.5-245846.0.) | |
| Chemokine (C-X-C motif) ligand 11 (CXCL11) | 25.5 (0.5-231.8) | 17.8 (0.5-154.7) | 32.2 (0.6-231.8) | 20.5 (0.5-154.3) | |
| Chitinase 3-like 1 (CHI3L) | 3004.0 (525.4-22288.0) | 2986.1 (545.1-22288.0) | 3004.0 (525.4-22091.1) | 3391.9 (115.0-17970.2) | |
| C-reactive protein (CRP) | 658.7 (36.7-12461.7) | 618.5 (37.9-8036.0) | 674.7 (36.7-12461.7) | 467.9 (17.1-11274.0) | |
| FMS-like tyrosine kinase 3 ligand (Flt3L) | 8.6 (0.1-44.9) | 7.0 (0.1-36.2) | 9.3 (0.1-44.9) | 9.0 (0.1-53.1) | |
| Interferon **α** (IFN-**α**) | 13.2 (0.4-101.0) | 12.8 (0.7-92.2) | 13.2 (0.4-101.0) | 15.1 (0.3-183.2) | |
| Interleukin 8 (IL-8) | 69.8 (20.1-566.0) | 56.1 (21.0-354.0) | 75.9 (20.1-566.0) | 62.2 (1.3-591.9) | |
| Macrophage colony-stimulating factor (M-CSF) | 81.6 (1.2-988.8) | 74.1 (1.2-922.4) | 94.2 (1.3-988.8) | 75.9 (1.0-1570.3) | |
| Platelet derived growth factor (PDGF-AA) | 84.2 (15.8-215.5) | 85.2 (46.7-205.9) | 81.8 (15.8-215.5) | 83.9 (0.8-278.8) | |
| S100 calcium-binding protein A8 (S100A8) | 221.9 (52.8-2930.8) | 246.8 (52.8-2248.1) | 215.1 (56.8-2930.8) | 285.5 (14.1-4655.2) | |
| TNF receptor superfamily member 9 (TNFRSF9) | 1.9 (0.2-48.8) | 2.5 (0.2-31.4) | 0.7 (0.2-48.8) | 1.7 (0.2-48.9) | |
| Vascular cell adhesion protein 1 (VCAM-1) | 1856.3 (19.5-15223.7) | 1429.3 (22.6-15223.7) | 2001.7 (19.5-12201.8) | 1449.2 (19.8-14643.8) | |
| Vitamin D binding protein (VitDBP) | 17643.7 (1254.6-98085.9) | 18380.7 (2552.2-75678.3) | 17643.7 (1254.6-98085.9) | 15325.4 (974.4-82571.0) | |

Data are presented as the median (min-max). GD, Graves' disease.

^*^pg per mL.

^†^Median value below lower limit of detection due to high proportion (˃50%) of imputed data.
